# Work System and Process Designs for Community Pharmacy-Medical Clinic Partnerships to Improve Retention in Care, Antiretroviral Adherence, and Viral Suppression in Persons with HIV

**DOI:** 10.3390/pharmacy8030125

**Published:** 2020-07-22

**Authors:** Jon C. Schommer, Oscar W. Garza, Michael S. Taitel, Osayi E. Akinbosoye, Sumihiro Suzuki, Patrick G. Clay

**Affiliations:** 1 College of Pharmacy, University of Minnesota, 308 Harvard Street, SE, Minneapolis, MN 55455, USA; 2 School of Clinical Sciences, College of Pharmacy, New Orleans Campus, University of Louisiana—Monroe, 478 South Johnson St, Suite 230, New Orleans, LA 70112, USA; ogarza@ulm.edu; 3 Walgreens Boots Alliance, 102 Wilmot Road, Deerfield, IL 60015, USA; michael.taitel@walgreens.com; 4 Astellas US, 1 Astellas Way, Northbrook, IL 60062, USA; osayi.akinbosoye@astellas.com; 5 Department of Biostatics and Epidemiology, School of Public Health and College of Pharmacy, University of North Texas Health Science Center, 3500 Camp Bowie Blvd, Fort Worth, TX 76107, USA; sumihiro.suzuki@unthsc.edu (S.S.); patrick.g.clay@gmail.com (P.G.C.)

**Keywords:** HIV, pharmacist, implementation, Patient-Centered HIV Care Model, pharmacy, viral suppression, retention in care, adherence, antiretroviral

## Abstract

The objective of this project was to collect and analyze information about work systems and processes that community pharmacy-medical clinic partnerships used for implementing the Patient-Centered HIV Care Model (PCHCM). Paired collaborations of 10 Walgreens community pharmacies and 10 medical clinics were formed in 10 cities located throughout the United States that had relatively high HIV prevalence rates and existing Walgreens HIV Centers of Excellence. Patient service provision data and most significant change stories were collected from key informants at each of the clinic and pharmacy sites over an 8 week period in 2016 and through in-depth phone interviews. Written notes were reviewed by two authors (J.C.S. and O.W.G.) and analyzed using the most significant change technique. The findings showed that half of the partnerships (n = 5) were unable to fully engage in service implementation due to external factors or severe staff turnover during the project period. The other half of the partnerships (n = 5) were able to engage in service implementation, with the most impactful changes being related to strong patient care systems, having a point person at the clinic who served as a connector between sites, and having pharmacists integrated fully into the health care team.

## 1. Introduction

In August 2014, the Centers for Disease Control and Prevention (CDC), Walgreens Corporation, and the University of North Texas Health Science Center implemented the Patient-Centered Human Immunodeficiency Virus (HIV) Care Model (PCHCM). A full description of the model has been published [1] and only elements pertinent to this analysis are included herein. The model provided pharmacist-led medication therapy management (MTM) [2] services combined with collaborative communication between clinic providers and community pharmacists. Paired collaborations of 10 Walgreens community pharmacies and 10 medical clinics (comprised primarily of HIV care providers) were formed in 10 cities located throughout the United States that had relatively high HIV prevalence rates and existing Walgreens HIV Centers of Excellence. 

During implementation of the PCHCM, clinics shared information with pharmacies including (1) complete medication lists, (2) medical problems lists, (3) lab test results (e.g., VL and CD4), (4) social history (e.g., tobacco use), and (5) immunization history. Pharmacies used this information to develop and implement patient services including (1) medication therapy management (MTM), (2) lab monitoring, (3) refill monitoring/retention of care, (4) medication adherence support, and (5) ancillary services (e.g., specialized packaging, delivery, and insurance consults). 

For the PCHCM, medication therapy management (MTM) was operationalized as “medical care provided by pharmacists whose aim is to optimize drug therapy and improve therapeutic outcomes for patients” [2]. For this project, MTM included performing patient assessment, targeted and/or comprehensive medication review, formulating a medication treatment plan, monitoring efficacy and safety of medication therapy, enhancing medication adherence through patient empowerment and education, and documenting and communicating MTM services to clinic providers (e.g., prescribers) in order to maintain comprehensive patient care. 

Part of the evaluation for the PCHCM since 2016 has focused on clinical and economic outcomes including retention in care [1], medication adherence [3], viral suppression [3], cost of service [4], and cost effectiveness [4]. With these evaluations completed, another important question to ask relates to the implementation of this program at each respective site. Each of the 10 pharmacy–clinic partnerships was allowed to develop and customize the communication and collaboration strategies that best applied existing strengths at their locations. Thus, the 10 pharmacy–clinic partnerships varied in terms of these characteristics and created an opportunity for further evaluation into implementation strategies at the dyadic level. Such information would be helpful for development of this patient care model in other settings. Thus, the objective of this specific analysis of the PCHCM was to describe the various work systems and processes that pharmacy–clinic partnerships used for implementation of a program that had the goals of improving retention in care, antiretroviral adherence, and viral suppression in persons with HIV. 

## 2. Materials and Methods

### 2.1. Program Evaluation Framework

The Program Evaluation Framework [5] was used for guiding the description and interpretation of implementation strategies. This framework affords the opportunity to understand how a program works—how it produces the results that it does. This perspective can provide recommendations for:

- Increasing the impact of the care model.
- Improving delivery mechanisms to be more efficient and reduce waste.
- Providing information for promoting the care model to communities and sponsors.
- Deciding which models should be retained or expanded.
- Replicating effective models elsewhere.

Within the Program Evaluation Framework [5] context, the Systems Engineering Initiative for Patient Safety (SEIPS) Model [6] was used for categorizing key characteristics of each clinic and each pharmacy that help describe the work systems, processes of care, and outcomes of interest for each site. This model was developed by the UW-Madison, Center for Quality and Productivity Improvement (http://cqpi.engr.wisc.edu/seips_model) and was successfully applied in similar evaluations of community pharmacy partnerships in the areas of hospice care [7] and cognitive pharmaceutical services [8].

Within that model, the individual (provider and/or patient) is at the center of “work systems” which should be designed to enhance and facilitate performance by the individual and to reduce and minimize the negative consequences on the individual (such as reduced stress) and therefore the organization (for example, improved organizational performance) [6]. Work systems relate to people, organizational coordination, technologies and tools, tasks, and overall work environment. “Processes of care” focus on how care is provided, delivered and managed. Thus, it is not just the patient care processes, but also includes things like information flow, purchasing and/or billing, reporting and monitoring [6]. Finally, “outcomes of interest” emphasize linkages between patient outcomes and employee/organizational outcomes [6]. For example, this may include job satisfaction, job stress and burnout, employee turnover, organizational health (profitability, meeting goals, changes in cost of care, waste reduction, decrease in need for re-work), patient safety, and quality of patient care (such as retention of HIV care, adherence, HIV viral load suppression, opportunistic infection, access to medications, access to information, changes in patient or caregiver knowledge, or clinical effect of medication recommendations).

### 2.2. Most Significant Change (MSC) Technique

With the Program Evaluation Framework in place and characteristics of the SEIPS Model defined, data were collected using the most significant change (MSC) technique [9]. The MSC technique is a form of participatory monitoring and evaluation that occurs during the program implementation cycle and provides information for describing work systems and processes that community pharmacy–medical clinic partnerships used for implementation and for describing key outcomes of interest. The most significant change (MSC) technique [9] asked about ‘significant change’ stories from those directly involved in the intervention, including the most significant change of all during the project period. The Appendix A contains the interview form and describes topics that were covered during structured interviews. 

### 2.3. Sample and Data Collection

The analysis sample included a key-informant employee from each of the 10 clinic and pharmacy locations. Key informants were selected by each site as the best representative for reporting information about the implementation of the PCHCM. Thus, a total of 20 key informants comprised the sample. Per-week reporting about the work systems, processes, and outcomes of interest was conducted over two, four week periods in 2016 (11 January–5 February and 1 August–26 August). In addition, each key informant participated in an in-depth interview that was conducted between 25 April and 13 May 2016 (the time period between the two, four week reporting periods) using the interview form in the Appendix A. Typically, interviews lasted approximately one hour. It should be noted that one interview (Clinic, Site 10) needed to be rescheduled due to time conflicts for the key informant. This interview was completed on 30 September 2016, which was after all other data reporting was completed.

### 2.4. Data Analysis

Written notes from weekly reporting and the in-depth interviews were reviewed by two authors (J.C.S. and O.W.G.). They reviewed the notes as a series of stories from the key informants and categorized the stories into work system, process, or outcome characteristics for each site. They focused on significant change stories and the most significant change overall that was reported by each key informant (refer to Appendix A). Selected stories were verified by continued communication with each key informant by the study team. As a final step, two authors (J.C.S. and O.W.G.) reviewed the findings from the 10 sites and came to a consensus about grouping the sites into descriptive themes using the Program Evaluation Framework [5].

## 3. Results

Key informants from each of the 10 partnerships provided weekly data and participated in phone interviews (n = 20). Table 1 summarizes key characteristics and the most significant change reported by each dyadic site. Based on key informants’ descriptions during interviews, the sites were grouped into four categories. First, three partnerships (01, 03, and 06) were described as “creation of work systems.” These partnerships developed explicit work systems based on existing strengths and created unique care models. For example, Site 01 used a triage approach coordinated by a nurse who then recruited, tailored and referred patients to suitable care services. Site 03 took advantage of the locational convenience between the clinic and pharmacy and developed a clinic-based appointment system that included pharmacist visits. Site 06 adjusted its communication technique so that physicians and pharmacists could collaborate using a virtual care model that included strong communication channels and full access to electronic medical records for all care providers. 

Second, two other partnerships (04 and 10) were described as “Team-Based Collaborative Care Processes” since they developed a collaborative process of care that incorporated pharmacist expertise into the overall team. The focus was upon processes that included the pharmacist as part of the care team. This was accomplished by having a liaison between the clinic and the pharmacy to develop links.

Third, two sites (07 and 08) were described as “Pharmacy Only-Driven Model Due to Staff Turnover at Clinic”. For these two sites, the most significant change related to staff turnover at the clinic. This resulted in the pharmacy site driving patient care during this time period. 

Finally, three sites (02, 05, and 09) were described as “Inaction” in that they were not able to make changes at the clinic or the pharmacy due to external factors. Unforeseen external factors that occurred in 2016 (evaluation period) were not able to be anticipated at the time of study enrollment (2013). An example of an unforeseen external factor relates to an individual who served as a champion for the project in 2013 but then accepted a position with another organization by the time the project started in 2016. Another example of an unforeseen external factor relates to a statewide policy that required medication services to be obtained through a contracted provider (negotiated for a statewide program). This policy change blocked patients from obtaining medication services from the pharmacies in this project. 

Overall, the most significant changes for three sites related to creation of new work systems. For two sites, the most significant change related to creation of team-based collaborative care processes. Another two sites needed to rely on the pharmacies to drive the model due to staff turnover at the respective clinics. Finally, three out of the 10 sites were unable to make significant changes due to unforeseen external circumstances such as significant personnel changes or state policy changes. 

## 4. Discussion

### 4.1. Application of Findings

The objective of this specific analysis of the Patient-Centered HIV Care Model (PCHCM) was to describe the various work systems and processes that community pharmacy–medical clinic partnerships used for implementation and to use these descriptions to make recommendations for future application. The findings showed that half of the partnerships (n = 5) were not able to fully engage in service implementation due to external factors (for example, statewide policy changes regarding preferred providers of medication services) or severe staff turnover (in particular, losing a project champion) during the project period. This supports the notion that, in addition to paying attention to work systems and processes (what to change) for implementing new models of patient care, it is important to consider organizational entrepreneurial orientation (readiness for change) and organizational flexibility (responsiveness for change) as new models are implemented [10]. Regarding work system design (what to change) [6,8] considerations related to personnel, task definitions, work space, tools/technology, and organizational culture, coordination, and communication are important. Entrepreneurial orientation (readiness for change) can entail an organization’s proactiveness, risk-taking, autonomy provided to employees, and style of work ethic [11]. Finally, an organization’s flexibility (responsiveness for change) can be viewed as being inflexible (steady state is desired), operationally flexible (short term orientation), structurally flexible (medium term orientation), or strategically flexible (long term orientation to address uncertainty) [10]. We recommend that these characteristics of organizations should be described and considered before implementing new programs.

The other half of the partnerships (n = 5) were able to engage in service implementation, with the most significant changes being related to the intentional creation of work systems and processes for care (see Table 1). Characteristics of the most impactful and efficient medical clinic and community pharmacy partnerships included having strong patient care systems in place, having a point person at the clinic who served as a connector between sites, and had pharmacists integrated fully into the health care team. It appears that successful pharmacy sites were intentional about transitioning from a performance-based approach to a relationship-based approach (with providers and patients alike). Developing well-planned work systems and processes of care that used existing strengths of the clinic and pharmacy were important as well. This required planning and investment in change, but achieved successful implementation. 

Regarding recommendations for the promotion, expansion and replication of the PCHCM, we suggest that careful assessment of aforementioned externalities that might interfere with an organization’s responsiveness for change, readiness for change, and stability of staff for championing change would be an important first step [6,8,10,11]. If those conditions are met, we then recommend the intentional investment in changing work systems and processes of care that build on existing and unique organizational strengths for implementation of the new patient care model. 

These shifts will transform community pharmacies to being organized by their capacity to operate as health care access points that provide, and are compensated for, patient care and public health services [12]. There is emerging evidence that comprehensive integrated care models are being created by pharmacies through integration with clinics, medical centers, and places of employment so that medication and medical costs can be combined in risk portfolios and meet pay-for-performance goals [13,14,15,16,17,18,19,20]. 

### 4.2. Limitations

The results, and our interpretation of them, should be tempered within the limitations of this project. First, the project objective focused on the various work systems and processes that pharmacy–clinic partnerships used for implementing the Patient-Centered HIV Care Model (PCHCM). It should be noted that these partnerships provided services to broader populations that were not included in our analysis. Second, other evaluations that showed improved retention in care, medication adherence, and viral suppression [1,3] were conducted for all of the 10 sites combined due to sample size considerations and were not site specific. Thus, those outcomes are not included for dyadic-level analyses presented in this article. Third, data were collected over an 8 week period and during one scheduled interview at each site during 2016. This approach does not account for seasonal or other time-related variation. Fourth, key informants for in-depth interviews were employees of each clinic or pharmacy site. There were no interviews completed with patients who received patient care services. Thus, we have no information about patient-reported outcomes or patient-reported experiences. Such information would provide an even more in-depth understanding of the provision of these services. Finally, only 10 sites were included for this demonstration project and only one community pharmacy organization was represented. It is likely that geographic and corporate ownership variation would affect the findings.

## 5. Conclusions

The objective of this analysis was to collect and analyze information the various work systems and processes that pharmacy–clinic partnerships used for implementing the Patient-Centered HIV Care Model (PCHCM). The findings showed that the most impactful and efficient medical clinic and community pharmacy partnerships had strong patient care systems in place, had a point person at the clinic who served as a connector between sites, and had pharmacists integrated fully into the health care team. 

## Figures and Tables

**Table 1:** Changes Reported by Each Site Categorized by Work System, Process of Care, or Outcome of Interest; and the Most Significant Change Overall.

Site Number	Work System	Process of Care	Outcome of Interest	Most Significant Change
	Creation of Work Systems			
01	Clinic: Triage by RN plus electronic messaging; RPh part of the team. Triage/Recruit/Refer/Tailor.		Pharmacy: Better pharmacist–patient relationships.	Triage by nurse plus electronic messaging with pharmacist who was added as part of the team.
03	Clinic: Pharmacist in the clinic area and a clinic-based appointment practice was developed that can be sustained. Pharmacy: Champion at clinic (an individual) helped create the needed connectivity.			Pharmacy: Champion at clinic (an individual) helped create the needed connectivity.
06	Clinic: New communication technique created too much paper work and inefficiencies. Went back to old way—MD and RPh communicate directly one on one through a virtual care model.		Pharmacy: We raised patient expectations. Sometimes could not meet those higher expectations and patients were dissatisfied.	Evaluation of new communication technique revealed inefficiencies. Went back to old way with physician and pharmacist communicated directly one on one, but now through remote communication.
	Team-Based Collaborative Care Process			
04		Clinic: Clinic staff person served as a liaison and developed collaborative care team so that pharmacist expertise was part of the care process.		Clinic staff person served as a liaison and developed collaborative care team.
10		Clinic: Collaboration—everyone working together. Pharmacy: Optimization of the patient care process. However, still need to make sustainable and develop documentation that is actionable.		Collaboration—everyone working together and optimizing it in the process of patient care.
	Pharmacy Only-Driven Model Due to Staff Turnover at Clinic			
07	Clinic: Severe staff turnover and change in software vendor caused a halt in project activity. Pharmacy: Clinic halted project activity. Pharmacy cared for patients on its own.			Severe staff turnover and change in software vendor caused a halt in project activity
08	Clinic: RN coordinator came in mid-stream. Tough transition. Pharmacy: Pharmacy is developing “complete care” models on its own.			RN coordinator came in mid-stream. Tough transition.
	Inaction			
02				Clinic: No significant changes. Pharmacy: No significant changes.
05				Clinic: No significant changes. Pharmacy: No significant changes.
09				Clinic: No significant changes. Pharmacy: No significant changes.

## Appendix A. Interview Form

Opening Question
  (1) Just to get us started, I’d like you to think about all of the work that is being accomplished at your practice site. What is the first thing that comes to mind?
Transition Question
  (2) Now, I’d like you to think about the Model of Patient-Centered HIV Care program that is being implemented at your practice site. What is the first thing that comes to mind?
Key Questions
  (3) Over the past six months, what changes related to **work systems** have been made at your practice site in order to implement the Model of Patient-Centered HIV Care program? This can include such things as: (a) personnel education, skills, knowledge, motivation, needs, (b) organizational coordination, collaboration and communication, (c) work schedule adjustments, (d) technologies and tools, (e) job workloads and protocols, (f) work area adjustments, or other related things. What worked well and what are areas for improvement?
  (4) Regarding work systems, what was the most significant change that occurred over the past six months?
  (5) Over the past six months, what changes related to work **processes** have been made at your practice site in order to implement the Model of Patient-Centered HIV Care program? This can include such things as: (a) Patient care process adjustments, (b) information flow adjustments, (c) purchasing and/or billing adjustments, (d) process improvement activities, (e) reporting and monitoring adjustments, or other related things? What worked well and what are areas for improvement?
  (6) Regarding processes, what was the most significant change that occurred over the past six months?
  (7) Over the past six months, what changes related to **outcomes** have been made at your practice site in order to implement the Model of Patient-Centered HIV Care program This can include such things as: (a) personnel job satisfaction, (b) job stress and burnout, (c) employee turnover, (d) organizational health such as profitability, meeting goals, changes in cost of care, waste reduction, decrease in need for re-work, (e) Patient safety such as drug therapy problem identification, complexity, or outcome, (f) quality of Patient care such as retention of HIV care, adherence, HIV viral load suppression, opportunistic infection, access to medications, access to information, changes in Patient or caregiver knowledge, clinical effect of medication recommendations, or other related things? What worked well and what are areas for improvement?
  (8) Regarding outcomes, what was the most significant change that occurred over the past six months?
  (9) From all of the significant changes you described, what do you think was the most significant change of all?
Ending Question
  (10) Thank you for helping us learn more about (1) changes that have been made at your site, (2) what worked well, and (3) areas for improvement relating to the implementation of your Model of Patient-Centered HIV Care. Finally, is there anything else that you would like to say?
